# Engaging Stakeholders in Identifying Access Research Priorities for the Department of Veterans Affairs

**DOI:** 10.1007/s11606-021-07195-5

**Published:** 2022-03-29

**Authors:** Demetria M. McNeal, Kelty Fehling, P. Michael Ho, Peter Kaboli, Stephanie Shimada, Sameer D. Saini, Bradley Youles, Karen Albright

**Affiliations:** 1 Division of General Internal Medicine, University of Colorado School of Medicine, Academic Office One, 12631 E 17th Avenue, Aurora, CO 80045 USA; 2 Denver-Seattle Center of Innovation for Veteran-Centered and Value-Driven Care, Rocky Mountain Regional VA Medical Center, 1700 N Wheeling St, Aurora, CO 80045 USA; 3 Iowa City VA Center for Access Delivery Research, Iowa VA Healthcare System, 601 Highway 6 West, Iowa City, IA 52246 USA; 4 VA Center for Healthcare Organization and Implementation Research, VA Bedford Healthcare System, 200 Springs Road, Building 70, Bedford, MA 01730 USA; 5 VA Ann Arbor Center for Clinical Management Research, VA Ann Arbor Healthcare System, 2215 Fuller Road, Mail Stop 152, Ann Arbor, MI 48105 USA

## Abstract

**Background:**

The Veterans Access Research Consortium (VARC), a Department of Veterans Affairs (VA) Consortium of Research focused on access to healthcare, has been funded by VA’s Health Services Research and Development Service (HSR&D) to develop a research roadmap for healthcare access. The goal of the roadmap is to identify operationally aligned research questions that are most likely to lead to meaningful improvements in Veterans’ healthcare access.

**Objectives:**

To describe the process of soliciting diverse stakeholder perspectives about key priorities on which VA’s HSR&D access agenda should focus and identify the results of that process.

**Methods:**

We used a modified Delphi approach to engage researchers and VA operational partners in a process to develop recommendations regarding the access-related research questions VA should prioritize. We then collaborated with three Veteran Engagement Groups (VEGs) across the country to solicit Veterans’ reactions to the Delphi results and their perspectives about access-related issues affecting access to VA health care.

**Results:**

The Delphi panel consisted of 22 research and operational experts, both internal and external to VA. The Delphi process resulted in five research questions identified by the panelists as highest priority for VA to pursue, each representing one of the following domains: (1) measurement of access, (2) barriers to access, (3) equity and subpopulations, (4) effective interventions to improve access, and (5) consequences of poor/better access. Veterans’ perspectives focused primarily on the barriers to access domain. Veterans indicated several barriers that might be addressed through research or operational initiatives, including poor communication about services, weak connections to and partnerships with local community care facilities, and poor provision of telehealth resources and education.

**Conclusions:**

Engaging multiple methods to solicit stakeholder perspectives enables more nuanced understanding of access-related priorities for VA. Future research should consider utilizing such an approach to identify additional research and/or operational priorities.

**Supplementary Information:**

The online version contains supplementary material available at 10.1007/s11606-021-07195-5.

## BACKGROUND

Access to health care has been identified as a critical issue by the Department of Veterans Affairs (VA) and the greater medical community.^1–5^ As defined by the original Institute of Medicine report that examined the relationships between access to care and sociodemographic factors (e.g., income, race/ethnicity, location), access is having “the timely use of personal health services to achieve the best health outcomes.”^6^ Fortney et al.^7^ have more recently emphasized the multi-dimensionality of access, conceptualizing it as spanning geographical, temporal, financial, cultural, and digital dimensions that together characterize the fit between the patient and the healthcare system. They also draw an important distinction between actual access to care (i.e., directly observable and objectively measurable dimensions of access; e.g., travel distance or time) and perceived access to care (i.e., self-reported and subjective dimensions of access; e.g., time convenience).^7^ Improving Veterans’ access to care across these multiple dimensions is essential to ensuring optimal health outcomes, and VA has been engaged in a number of recent initiatives to investigate and address barriers to access. Such initiatives include the Veterans’ Access to Care through Choice, Accountability, and Transparency Act (Veterans Choice Act) of 2014 and the VA Maintaining Internal Systems and Strengthening Integrated Outside Networks (MISSION) Act of 2018,^8,9^ which were enacted to improve Veterans’ access to healthcare, including enhancing in-network and non-VA healthcare access, access to urgent care in the community, and authorization of telehealth across state lines.

The VA’s Health Services Research and Development Service (HSR&D) supports research that encompasses all aspects of VA healthcare, focusing particularly on patient care, cost, and quality. HSR&D’s mission involves making research relevant to a range of stakeholders, including operational partners and Veterans. As an intramural program unique to VA, HSR&D operates under a research model that emphasizes close partnership with clinical and policy stakeholders within VA and translating research findings into broader implementation within a learning healthcare system (LHS). The Veterans Access Research Consortium (VARC), a VA Consortium of Research focused on access to healthcare, was funded by HSR&D in 2020 to leverage the LHS framework to accelerate access-related health service research, build a community of researchers with the goal of improving access to care for Veterans, and enable a more comprehensive understanding of access-related concerns from both research and operational perspectives.

A key part of VARC’s mission is to develop a roadmap to guide VA toward research likely to lead to meaningful improvements in Veterans’ healthcare access. In February 2020, VARC developed and administered the ARC Network Needs Assessment Survey to the HSR&D research community to help determine the specific direction of VARC activities.^10^ Among VARC’s charges in its first year was identification of key priorities on which HSR&D’s access research agenda should focus in the coming years. In order to accomplish this, VARC sought input from multiple stakeholders, including access researchers from within and outside VA, VA operational partners, and Veterans. This manuscript describes the process of soliciting the diverse perspectives of researchers, operational experts, and Veterans around priorities for access research and the key priorities identified as a result of that process.

## METHOD

### Study Design

To understand access-related priorities from the perspectives of multiple stakeholders, we designed a two-step methodological approach. We first sought to identify research questions that experts in the field of access research believed to be most important to answer in order to advance VA’s goal of improving access to healthcare and, ultimately, improving health outcomes for all Veterans. Experts for our purposes included researchers with expertise in the field of healthcare access, including both those who work within VA and are familiar with its history of approaching access-related issues and those who work primarily outside VA but who have expertise in military systems science and/or Veteran health challenges, as well as VA operational partners who head offices within the VA which typically fund access-related initiatives. The vantage points of such operational partners’ institutional history and social location allow them a deep understanding of the gaps in VA work to date and system-level access needs and barriers that remain ripe for further investigation.

In order to build consensus and facilitate dialog across these researchers and operational partners, we utilized a modified Delphi method. The Delphi method is a structured, iterative process through which a group of experts and stakeholders reach a consensus on a particular topic through a specific number of rating rounds that integrate controlled feedback.^11^ The rounds involve a dynamic process of data gathering and analysis, throughout which participants rethink and modify their opinions. The Delphi process is a low-cost and adaptable procedure that has previously been used to successfully reach consensus on a wide variety of health-related topics,^12,13^ making it an optimal method to secure expert input regarding access-related priorities relevant to VA. Our modified approach involved the addition of a final, synchronous discussion round in addition to multiple structured rating rounds.

We then sought to solicit Veteran input on the results of the Delphi process, particularly whether/how they perceived the areas of focus identified by the researchers and operational partners to be relevant to their own experiences and whether there were specific aspects of those areas that they regarded as a particularly high priority. In intentionally soliciting such Veteran input, we followed what has increasingly come to be regarded as a best practice within VA. The concepts of Veteran engagement and patient-centered care have become key drivers in VA efforts to redesign health care and, in recent years, HSR&D has increasingly emphasized engaging Veterans as partners in research.^14^ Indeed, engaging Veterans in the development, implementation, and analysis of VA research is strongly recommended in order to strengthen understanding of data and ensure a focus on useful and meaningful research questions that can improve VA health services and the care it provides.^15^.

We chose to solicit Veteran perspectives as a discrete second step in our method, rather than simply include Veterans in the Delphi process, because we believed that having a diversity of Veteran voices engaged in in-depth conversations in familiar and localized settings would be more likely to support and promote honest and informative feedback.^15^ We did not want to risk having Veteran perspectives potentially drowned out by those of the research and operational partner Delphi participants, and thus miss an opportunity to better understand Veterans’ lived experiences. VEGs provided an excellent opportunity through which to engage Veterans in focused discussion. The explicit mission of each VEG is to provide a forum in which Veterans contribute their perspectives and input on VA research studies. VEGs are typically comprised of Veterans partnered with researchers at VA HSR&D–funded Centers of Innovation affiliated with particular VA Medical Centers. Though specific structure and format may vary, they typically gather monthly to quarterly and meet for 1–2 h, during which research teams present their proposals or results for Veteran feedback.

### Delphi Process

Purposive sampling was used to identify experts in access-related research across the three aforementioned categories: (1) researchers conducting access-related research within the VA; (2) researchers conducting access-related research outside the VA but with expertise in Veteran populations; and (3) operational partners in leadership roles within VA offices whose missions are aligned with improving Veteran access to healthcare. Experts were identified through searches of the academic literature on healthcare access, identification of participants in conferences addressing healthcare access, and recommendations through VARC professional networks. Efforts were made to identify experts working across a variety of geographic regions and substantive topics within access research. Thirty-one experts (11 VA researchers, 10 researchers external to VA, and 10 operational partners) were initially contacted via email to participate. Twenty-two (9 VA researchers, 4 researchers external to VA, and 9 operational partners) ultimately accepted the invitation, agreeing to participate in the Delphi process and work toward the development of a consensus regarding high-priority access-related research questions. A list of participants’ affiliations and areas of expertise is presented in Appendix A in the Supplementary Information.

**Figure 1:**
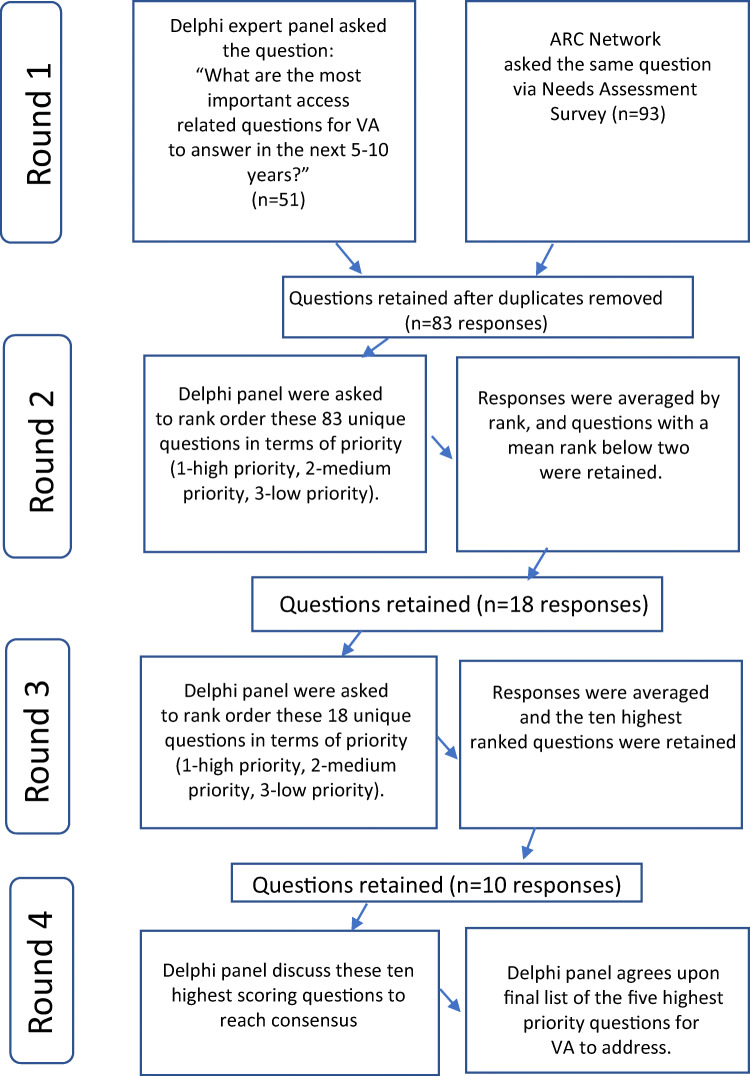
Flow diagram of modified Delphi process.

The Delphi process is depicted in Fig. 1. Throughout July and August 2020, Delphi participants responded to three rounds of emails soliciting their perspectives about the most important access-related research questions for VA to address. The first round asked each participant to provide at least two responses to the open question: “What are the most important access-related questions for VA to answer in the next 5–10 years?” Fifty-one responses were received (Appendix B in the Supplementary Information). These were then compared with responses to the same question that had been asked in the ARC Network Needs Assessment Survey.^10^ Sixty-six survey respondents had responded to this optional question, together offering a total of 93 answers, some of which were duplicative (Appendix C in the Supplementary Information). After the 51 research questions suggested by Delphi panelists were merged with the 93 research questions suggested by ARC survey participants, 61 duplicates were eliminated, ultimately resulting in a final set of 83 unique research questions (Appendix D in the Supplementary Information).

In the second round, Delphi participants were asked to rank order these 83 unique questions in terms of priority (1—high priority, 2—medium priority, 3—low priority). Responses were averaged by rank, and questions with a mean rank below two were retained (*n* = 18). In the third and final round of email engagement, participants were asked to identify their top ten highest priority research questions from among these 18. Rank scores were averaged and the ten highest-ranked questions were retained. In September 2020, Delphi panelists then participated in a fourth round of engagement, convening via a virtual videoconferencing platform to discuss these ten highest-scoring questions and to identify through consensus a final list of the five highest priority questions for VA to address. The 4-h discussion was facilitated by members of the VARC team (KA, DM) and included large-group discussion as well as breakout groups focused on specific substantive domains. Throughout the meeting, discussion took place both verbally and via the chat function embedded in the virtual platform.

### Veteran Engagement Groups

Following the virtual meeting, results of the Delphi panel were presented to three VEGs to solicit Veterans’ perspectives on the five research questions identified as highest priority by access researchers and VA operational partners. In October 2020, results were presented at a virtual meeting of the Center for Access and Delivery Research and Evaluation (CADRE) Veteran Engagement Panel in Iowa City, IA. In November 2020, results were presented virtually to members of the Center for Healthcare Organization and Implementation Research (CHOIR) Veteran Engagement in Research Group, in Bedford/Boston, MA. Finally, in November and again in December 2020, results were presented at two virtual meetings of the Center for Clinical Management Research (CCMR) Veteran Research Engagement Council, in Ann Arbor, MI. We were invited to a second meeting with the CCMR VEG because the Veterans enjoyed the robust conversation and wanted to continue the discussion. We do not report characteristics (e.g., race/ethnicity, age, branch of service) of VEG participants because they are not available; consistent with their desire to respect Veterans’ privacy and engage a Veteran-centered approach, VEG liaisons do not collect and/or release such information.

Each VEG presentation included an explanation of goals for the session, VARC’s mission to improve Veteran access to healthcare, and HSR&D’s goals and intention to provide optimal care to Veterans; a description of the Delphi process and results; and then a discussion in which Veterans offered their perspectives on their access-related experiences and on the relevance of the Delphi results. Following best practices for Veteran engagement^14^, and to facilitate a discussion that would be meaningful and relevant for Veterans, the questions guiding the discussion were primarily specific and practical (see sample questions in Table 1) rather than broad, vague, or overarching (e.g., “What are your reactions to this list?”). Notes were taken throughout the meeting to capture Veteran perspectives and were then triangulated with data provided by Veterans during the meeting via the virtual platform’s chat function. The triangulated data were then analyzed for thematic content using established content analysis methods.^16–18^.

**Table 1:** Sample of Questions Used in VEG Discussions

Access research domains	Discussion questions
Barriers to access	How do structural, logistic, personal, and organizational barriers to access vary across subpopulations and interfere with Veterans getting the care they need and/or desire?
	What are some of the things that you think act as barriers to access to care through the VA? Why or how are they barriers?
Equity across Veterans	How can we ensure equitable and effective access to services for Veterans who are underrepresented or experience disparities in the VA?
	Do you think there are differences in access to care across the Veteran population?
	What are those differences and why do you think those differences exist?
	Which differences should be addressed immediately?
Effective Interventions	What are the most effective and scalable interventions that improve access, considering different modalities (e.g., in person, virtual care), settings (e.g., VA, community), and targets (e.g., patients, providers, system)?
	What ideas do you have for how access issues within the VA might be addressed?

## RESULTS

The researchers and operational partners engaged in the modified Delphi process identified five research questions as the highest priority for VA to answer in the coming 5–10 years. These questions were categorized by participants as spanning five research domains, including the following: (1) measurement of access^19–21^ (“How should actual and perceived access be defined and measured so it is understandable, uses the best possible data, and has meaningful implications for Veteran outcomes, both in VA and the community?”); (2) barriers to access^22–24^ (“How do structural, logistic, personal, and organizational barriers to access vary across subpopulations and interfere with Veterans getting the care they need and/or desire?”); (3) equity and subpopulations^25–27^ (“How can we ensure equitable and effective access to services for Veterans who are underrepresented or experience disparities in the VA?”); (4) effective interventions to improve access^28–30^ (What are the most effective and scalable interventions that improve access, considering different modalities, settings, and targets? How does this vary for subpopulations?”); and (5) consequences of poor/better access^31–33^ (“Does increased access and/or better access lead to improved quality care coordination, patient satisfaction, clinical outcomes, care continuity, and cost? What are the systemic consequences?”). The final list of research questions and their corresponding domains are presented in Table 2.

**Table 2:** Final Results of Delphi Panel

Access research domains	Leading research questions for each domain
Measurement of access	How should actual and perceived access be defined and measured so it is understandable, uses the best possible data (surveys, electronic, etc.), and has meaningful implications for Veteran outcomes, both in VA and the community?
Barriers to access	How do structural, logistic, personal, and organizational barriers to access vary across subpopulations and interfere with Veterans getting the care they need and/or desire?
Equity and subpopulations	How can we ensure equitable and effective access to services for Veterans who are underrepresented or experience disparities in the VA (e.g., racial/ethnic minorities, LGBTQ, women, those living on tribal lands, etc.)?
Effective interventions to improve access	What are the most effective and scalable interventions that improve access, considering different modalities (e.g., in person, virtual care), settings (e.g., VA, community), and targets (e.g., patients, providers, system)? How does this vary for subpopulations?
Consequences of poor/better access	Does (a) increased access and/or (b) better access lead to improved quality care coordination, patient satisfaction, clinical outcomes, care continuity, and cost? What are the systemic consequences?

The subsequent discussions with Veterans in the VEGs focused almost exclusively on barriers to access. Three primary themes emerged from these discussions that provide more depth to this domain than that which could be produced by the Delphi panel, highlighting real-world barriers to access observed or experienced by many Veterans. First, Veterans repeatedly described a desire for better communication about services available to Veterans. These Veterans described a belief that information about how (i.e., process, location) to access services and resources are not disseminated effectively across the VA system, often resulting in the perception of inequitable or uneven distribution of such services or resources across regions. VEG participants stressed the need for widespread marketing and consistent messaging as well as more individualized contact in order to reach Veterans and inform them about what services were available, under what conditions, and points of contact for interfacing with the system. As one Veteran put it, “This is like showing where the doors to service are. If you don’t show people where the door is, there’s no access!”.

A second theme identified from VEG discussions was the need to build and/or strengthen connections to and partnerships with local community care facilities in order to improve access. Participants noted that many Veterans receive care through private health care organizations as well as through the VA and that better communication and coordination between the two is necessary to reduce record scatter, focus resources, and ultimately improve patient service and experience. The following dialog illustrates some of these concerns:

Veteran 1: I think we need to find a way before they are discharged [from VA facilities] to connect [with community care facilities].

Veteran 2: Veterans need truly informed consent about ALL options to even know what they want.

The need to improve connections to a variety of local community care facilities, including not just health care but also social service agencies, was also emphasized. In the concurrent chat discussion during one VEG meeting, a Veteran wrote the following:

Another thought that comes to mind as a barrier to access is the limited visibility and connection that VA has in local communities. It’s like a well-kept secret… community agencies know nothing about VA and what services are offered. AND VA representatives often don’t show up to community meetings (e.g., local homelessness strategic planning groups organized by cities or towns).

A final theme identified from analysis of VEG discussions was the importance of better provision of resources, including education and skill development, in order to truly enable telehealth to fulfill its potential to improve access. Veterans observed that, while VA was advanced in telehealth development, Veterans’ individual technological skill sets and access to equipment varied significantly. They emphasized the need for more education about how to use technological resources and available options for telehealth care, and perhaps even the distribution of technological devices (e.g., tablets) that would enable access to telehealth care for Veterans who would be otherwise unable to connect technologically. As one Veteran explained in a comment within the chat function,

[Telehealth] is a shiny object if [Veterans] can’t use the devices. The Veterans at [a local Soldier’s Home] received iPads from donors to assist with telehealth as well as reduce isolation from social distancing. However, many were not comfortable with the technology as many of them still have flip phones... the technology HAS to be paired with training.

## DISCUSSION

The two-pronged methodological approach utilized in this study highlights the different perspectives of multiple stakeholders concerned with issues related to access to VA healthcare, spanning both individuals who help to shape the state of knowledge about access and those who are affected by the implementation of that knowledge. The findings are intended to inform the agenda for access-related research and operational efforts and serve as a guide for resource allocation toward such efforts in the future. In parallel, VARC has also been conducting a systematic review of the VA access portfolio, including both funded research and operational projects related to access to care. A key next step will be to identify gaps in this portfolio in each of the high-priority areas identified through this process.

To our knowledge, this is the first use of a modified Delphi method to explore access-related priorities within VA. By involving researchers both within and outside VA, as well as VA operational partners, we were able to engage a diverse group of experts with knowledge of the history of and gaps in the field and insights into the opportunities and constraints of particular directions for future inquiry. Through participation in the Delphi process, these experts reached a consensus regarding the five highest priority research questions that should be asked and answered if VA is to achieve its goal of improving Veterans’ access to healthcare, thus achieving a critical step in VARC’s mission to develop a research roadmap to help guide VA in that effort. The research questions and domains identified by the Delphi panelists will be used to inform future HSR&D funding opportunities.

Engaging Veterans’ perspectives about access to healthcare via focused VEG discussions shed additional light on one of the domains prioritized by experts, thus complementing and deepening the Delphi results. Veteran input focused primarily on barriers to access, as they drew on their own and fellow Veterans’ direct experiences and perceptions. They offered less insight into the other domains identified by the Delphi participants. However, the Veteran perspective offers a personal granularity to the perceived barriers that the researchers and operational partners could not, highlighting key issues in patient experience that, if addressed, would significantly improve the patient-system interface.

The insights provided by Veterans suggest that their engagement with the health care system is limited by what they experience as poor communication regarding the services offered by the greater VA health care system. Veterans discussed how their lack of awareness of available services served as a significant barrier to access. Their perspectives indicate how this knowledge gap can impede care. Veterans also reported a perception that access to services is distributed inconsistently or and/or inequitably across the system. This perception, of course, may also be a result of poor communication. Such communication issues have been reported elsewhere as well. For example, a study seeking to identify Veteran-centric barriers to mental health care found that Veterans faced challenges navigating VA benefits and healthcare services which were related to lack of understanding them.^2^

The need to strengthen connections to and partnerships with local community care facilities in order to improve access was also recognized by Veterans. This corresponds to recent literature highlighting a need for strong coordination and communication between health care providers inside and outside VA, as Veterans are typically burdened with coordinating their own care when such resources are absent.^34^ However, efforts have been made to forge partnerships between communities and VA centers to create programs that reduce the burden of coordinating and accessing care. For example, the Mental Health-Clergy Partnership Program was established to develop programs to assist rural Veterans with mental health needs and reduce the stigma of seeking treatment.^35^ Additionally, the creation of weekly “Veteran Coffee Socials” allows Veterans to form relationships with each other, representatives from community organizations, and staff from local and VA healthcare resources. One of the most common activities of these socials involves receiving information and directions for enrollment into needed healthcare supports and to local community resources.^36^.

Veterans’ feedback also revealed that simply providing the technology to reduce geographical barriers to access is not sufficient for improving access. Instead, they stressed the need for training about how to actually use the technological resources provided and education about the available options for telehealth care. This is an insight consonant with a recent study evaluating Veterans’ experiences with VA-issued tablets to identify patient characteristics associated with preferences for video visits compared to in-person care.^37^ Qualitative analysis revealed Veterans viewed telehealth as an opportunity to overcome access barriers but also noted the need for VA to “give a class on how to use the tablet and make sure the connection and passwords are done right.”

While researchers and operational partners who participated in the Delphi panel conceptualized barriers to care primarily as structural, logistical, and organizational issues, Veteran feedback described more fundamental needs: the need for better communication, both about available services generally and about how to use the technological tools that could support improved access via telehealth, and the need to develop inter-institutional relationships that could facilitate better connections across systems. Both sets of perspectives are instructive and should be incorporated into future research and operational efforts to improve Veterans’ access to healthcare.

### Limitations

Several limitations should be considered when interpreting these results. As with most Delphi processes, our approach involved a relatively small number of participants and the reliability of its results is impossible to determine. Among Delphi participants, there were fewer experts external to the VA than internal VA researchers and operational partners; however, we believe this weightedness is appropriate given the importance of deep understanding of VA processes, population, and history in identifying VA needs and future directions. We also report insights from Veterans enrolled in VA healthcare services, which may not reflect the perspectives of Veterans who have not used VA benefits and services. In addition, the VEGs engaged in this study were located in urban settings across three states, limiting the generalizability of the results across Veterans living in different regions and/or in rural areas. Our inability to report the characteristics of the Veterans who participated in these particular VEG discussions further limits generalizability. It is also important to remember that, although access has been identified as an issue of critical concern by VA and the greater medical community, our results cannot ascertain the relative merit of addressing access-related priorities versus other issues relevant to Veterans and VA research.

## CONCLUSION

Our approach is novel in its application of the modified Delphi method to engage researchers and operational partners in identifying access-related research questions to guide the focus of future HSR&D efforts to improve Veteran care. Our subsequent engagement of Veterans for feedback on those questions complements and deepens the results of that process by highlighting the relevance of one particular domain and generating insights into the specific ways that barriers to access may be experienced by Veterans. The researchers and operational partners engaged in the Delphi process identified five broad research questions on which HSR&D should focus in the coming decade, and subsequent engagement with Veterans provided more granular insight into particular barriers to access which might be fruitfully addressed operationally. Engaging directly with Veterans allows deeper understanding of how they experience health care, perceive research, and see the world through a unique lens shaped by military experience. This mixed-method approach to stakeholder engagement suggests a more comprehensive understanding of the diverse perspectives about access to health care and contributes to a relevant and timely discussion of the most critical domains for research effort and operational investment.

## Supplementary Information

Below is the link to the electronic supplementary material.

Supplementary file1 (PDF 329 KB)
