# Supplementary material for: Engaging Stakeholders in Identifying Access Research Priorities for the Department of Veterans Affairs
Source: J Gen Intern Med. 2022 Mar 29;37(Suppl 1):14–21. doi: 10.1007/s11606-021-07195-5 (PMC8993958; doi:10.1007/s11606-021-07195-5)
Supplement: Supplementary file 1 — Supplementary file1 (PDF 329 KB) [file 11606_2021_7195_MOESM1_ESM.pdf]

Appendix A. Affiliations and expertise of Delphi panelists

Panelist	Affiliation	Area of Expertise
1	VA Office of Veterans Access to Care (OVAC)	Access to Health Care, Specialty Care
2	Durham VA and Duke University	Care Coordination, Interventions
3	RAND Corporation	Military Mental Health, Health Technologies
4	VA Office of Mental Health & Suicide Prevention	Mental Health
5	University of Toronto and Sinai Health System & University Health Network	Health Policy
6	VA Office of Primary Care Operations	Primary Care
7	Puget Sound (Seattle) VA and University of Washington	Geographic Disparities, Telehealth
8	VA Office of Community Care	Non-VA Care in the Community
9	VA Office of Connected Care	Telehealth modalities
10	Portland VA and Oregon State University	Non-VA Care in the Community
11	VA Office of Specialty Care Services	Specialty Care
12	VA Office of Rural Health	Rural Health
13	VA Office of Health Equity	Health Equity
14	Boston VA and Boston University	Health Policy
15	Little Rock VA and University of Arkansas for Medical Sciences	Mental Health
16	University of Cambridge	Quality of Care, Interventions
17	VA Office of Mental Health	Mental Health
18	RAND Corporation	Military Health Policy
19	Portland VA and Oregon Health & Science University	Mental Health
20	Salt Lake City VA and University of Utah	Non-VA Care in the Community
21	Los Angeles VA and University of California - Los Angeles	Equity, Disparities
22	Palo Alto VA and Stanford University	Telehealth

Appendix B: Access-Related Research Questions Identified in Round 1 of Delphi solicitation (n=51)

How do we identify psychometrically validated instruments to measure Veteran's perceived access to care and develop methodologies to collected data about perceived access to care from a representative sample of Veterans who need treatment?
How do we identify what access barriers are interfering the most with Veterans getting the care they need?
What are the unintended consequences of using telehealth to address access to care – what are the unintended consequences?
Does telehealth/virtual care exacerbate disparities?
How do we create a learning health lab specifically focused on access to care?
When can telehealth be used as a substitute for in-person care?
When does purchasing care in the community improve access to timely, high-quality care for Veterans?
How can we expand access to medical and social services through telemedicine and other technologies while ensuring that Veterans receive care that is at the same or higher quality than they would in person?
How can we ensure equitable access to services for Veterans who are underrepresented or experience disparities in the VA?
How best to measure Veteran access to care (e.g., survey items, admin data, important covariates)?
How best to utilize technology to improve access to care (e.g., televideo, wearables)?
How best to match access interventions with Veteran needs, barriers, and resources?
What other metrics or outcomes does access to care impact (e.g., trust, satisfaction, economic)?
Is VA a health care system or an insurance provider? And how does VA address the split between providing community care and care within VA itself?
What are ways to decrease no-shows, make enrollment easier, and other make access more efficient and easier for Veterans?
In what ways can VA support, and potentially engage in treatment, Veterans who do not or have not used VA recently?
How best to use administrative data to measure the access experience of patients receiving care through non-traditional channels, including virtual care, telephone care, secure messaging, and e-consults?
How to optimize patient flows between primary and specialty care to manage overall access and quality of care?
How can continuity of care best be maintained or improved when initiatives are introduced to improve access?
For which VA patient groups is continuity of care most important?
How are the various dimensions (the 5 A's) of access related to outcomes and value for Veterans?
What are some of the most effective ways to reduce barriers to help seeking and retention for Veterans with health-related needs?
How can we most effectively reduce disparities for all Veterans? That is, what solutions for increasing access need to vary by subpopulation of Veterans (e.g., racial/ethnic minorities, LGBTQ, women, those living on tribal lands, etc.).
What are Veteran perceptions about what's close enough/timely enough care within VA and in community?
How do we design disease specific and patient-risk specific access measures to better represent the heterogeneity of access needs rather than blanket designations?
How do we expand technology to leverage access?
How should "access" be defined and measured in the VA and in the community?
Should timeliness of care be a component of "access" and if so how?
What technology exists or should be created to aide in answering these questions and implementing the answers?
How does virtual care impact access?
What are the best overall metrics to determine "good access"?
What is the best model to manage for Veterans to choose VA versus. care going to the community for care?

How does VA plan on mitigating the expected loss of roughly 60% of physicians and roughly 50% of APRNs as primary care providers over the next ten years?
How will virtual care play a role in improving access in primary care in the future?
How will artificial intelligence play a role in improving access in primary care in the future?
How do we define how VA will approach access to care? (It is not just about number of scheduled appointments)
How do we outline changes needed to continue to enhance access beyond the "provider just work more" mindset?
How will VA be able to hire qualified providers (physicians and APRNs) with current pay limitations?
What are the needs of our aging Veteran population and is VHA appropriately preparing to provide care to that population group?
How will social determinants of health impact VHA's ability to provide correct and appropriate care to Veterans?
Are access points in community partnership facilities (e.g., Wal Mart clinics, VERSUSO telehealth sites, libraries) a solution looking for a problem?
Is there in fact an explicit need for the expansion of services to remote locations?
If there is insufficient demand, is there political or public relations value in creating them?
What does past meager usage of such sites (telehealth to campuses, etc.) have to teach us about the future?
Is there or will there be enrollment or usage impact? If you build it, will they come? If yes, will new ones come, or will we simply be splitting existing VA patient panels and putting VA facilities in danger?
Are there specific groups of eligible Veterans who are less likely to access VA services and could targeted outreach to these groups ensure that they understand their options for accessing VA services?
Are there specific groups of enrolled Veterans who encounter barriers to care (e.g., language, literacy, transportation, lack of telehealth capacity, discrimination, harassment at facilities) and could target interventions to enable these groups to overcome these barriers?
Are there specific groups of enrolled Veterans who have difficulty adhering to VA treatment recommendations and could improve access to care coordination services enable better adherence?
How do we identify a process and interventions that can be reproduced and used in different contexts to understand barriers to access, but more importantly, reduce them?
How do we combine measures from more traditional channels to characterize overall access separately for new and established patients?
What are factors that influence the metrics around drive times and wait times?

Appendix C: Access-Related Research Questions Identified Through the ARC Survey (n=93)

How do we measure impact of the CHOICE and MISSION acts in improving access and/or quality of care for Veterans?
How to identify the biggest problems in access?
How to evaluate the effectiveness of current interventions (e.g., community care, telehealth) to improve access?
Is access effective? Does it lead to good outcomes broadly defined?
What do vulnerable Veterans need to help them with access that is effective?
Does increased access equal improvement, as demonstrated through better access, care quality, care coordination, patient satisfaction/preferences and manageable costs.
How can we improve actual and perceived access to care for patients who are struggling to manage chronic conditions?
How do we compare access to care and outcomes-VA versus non-VA care, after implementation of VA Mission act?
How can we best support smooth transitions across community and VA care providers?
How can we ensure access to high quality care for vulnerable Veteran groups (racial/ethnic minorities, women, LGBTQ, rural)?
How do we increase access to specialty care services without widening access disparities between urban/suburban and rural Veterans?
How can we ensure Veterans who are not currently accessing VA services due to distance from a VA facility, homelessness, or other factors are able to obtain quality care?
How does VA Video Care work in select populations - older Veterans, rural Veterans, and women Veterans? What is the quality of care provided for these groups?
How do we increase access and engagement in high quality, evidence-based behavioral health services?
How do we provide access to appropriate community services that are BEST delivered by the community and retain services BEST delivered by VA?
How we improve Veterans access to care that is evidence-based and high quality versus just focusing on temporal access?
How to increase quality access to care while the proportion of health providers are decreasing, and the population is growing older and sicker?
How do we improve access to evidence-based mental health care? How do we improve access to high quality guideline concordant mental health care provided in primary care? What novel care delivery methods improve access while maintaining quality?
Can VA improve value by reducing low value care?
How can we decrease use of low-value services to improve access to high-value care? How can we best make use of telemedicine to substitute for, rather than complement, in-person services? What are effective models to coordinate care received in VA and non-VA settings?
What is the correlation between resource utilization and outcomes in VA and non-VA settings?
How can we establish good metrics and evaluate policies for cost-effectiveness/outcomes?
How to provide Veterans with timely access to high-quality care that is coordinated across providers and systems?
How do we create timely access to the appropriate level of care needed? How do we create a scalable appointment scheme for patient access to match time needs?
How do we most efficiently and appropriately match a patient with different access options given their clinical needs and preferences? How do we best ascertain when it is important for a Veteran to be seen ASAP (whether that be within VA or in the community) and when things can wait instead of relying on arbitrary wait times?
When Medicare is expanded to everyone, what effect will that have on access and general system burden improvement?

How can we ensure access to high quality care for vulnerable Veteran groups (racial/ethnic minorities, women, LGBTQ, rural)?
How has the Mission Act changed access to care for racial/ethnic diverse Veterans who tend to be have less socioeconomic resources?
What is it about how access to care is currently structured that makes it a challenge for some Veterans to get those services?
How can we improve access to gender-sensitive, integrated health care for women Veterans in the VA--and work with community partners to advance health care for women Veterans?
Given the shift in the care, are VA's changing their organizational staffing and structure to meet the needs of Veterans?
How do we ensure equitable access to care for vulnerable Veteran populations (e.g., racial/ethnic minorities), particularly in the age of the MISSION Act?
How can we ensure Veterans who are not currently accessing VA services due to distance from a VA facility, homelessness, or other factors are able to obtain quality care? What actionable steps can we do to reduce disparities in access to care for underserved proportions of the Veteran population?
How does VA Video Care work in select populations - older Veterans, rural Veterans, and women Veterans? What is the quality of care provided for these groups?
How do we link and sustain linkage to care for highly vulnerable Veterans, such as those with or at risk for HIV, hepatitis, substance abuse, and homelessness?
How do we increase access to specialty care services without widening access disparities between urban/suburban and rural Veterans?
How do we understand tradeoffs and determine which services to support and under what conditions: outpatient care, homecare, tele-health, and community-based care, dependent on a Veteran's health and sociodemographic?
How do we engage non-VA Veterans? How do we maintain engagement in care for newly engaged Veterans? How do we engage recently discharged Veterans (e.g., DoD-->VA)?
Who are we losing (i.e., is not getting access), where are we losing them, and why?
How do we improve data collection on LGBT Veterans, this will allow us to answer questions about health disparities, and what access issues remain?
How do we improve access to care in remote areas? How do we use information technology to increase access? How do we improve communication tools and information systems to support care anywhere?
How can we improve telehealth access/availability?
How can we leverage technology tools to improve access to timely care, especially mental health?
How can we improve access by leverage social systems including family, friends, and community?
How can we address the acute shortage of mental health providers and care nationally?
How technology can be used to increase access for the most vulnerable Veterans (older, rural, homeless, etc.)
How to improve access to VA care for Veterans with visual impairment and for Veterans with visual dysfunction related to traumatic brain injury?
How do we identify population-specific access preferences to implement a flexible system that meets the access demands of the diverse Veteran population?
How do we optimize access that includes VA and non-VA care?
How do we determine what the VA builds (provides) or buys (community care)? What are developed and enhanced effective VA/community collaborative care models?
When is and isn't Community Care a good alternative to VA-delivered care?
How do we provide access to appropriate community services that are best delivered by the community and retain services best delivered by VA?
How can we understand the tradeoffs and determine which services to support and under what conditions between VA and community care (including commercial retailers)?
How can we optimize Veteran decision-making about use of VA and non-VA care? What are effective models to coordinate care received in VA and non-VA settings?

How can we better coordinate VA Community Care and VA Medical Facility care?
How can we coordinate care and improve communication between VA and non-VA providers?
How do we know whether the interventions already in place (e.g., community care, telehealth) are improving access?
What is the impact of the MISSION Act, especially on rural and other at-risk Veterans?
How the Mission Act has changed access to care for racial/ethnic diverse Veterans who tend to be have less socioeconomic resources?
Did the CHOICE and MISSION Act improve access and/or quality of care for Veterans? And if so, at what cost?
What is the correlation between resource utilization and outcomes in VA and non-VA settings?
How can we best support smooth transitions across community and VA care providers?
How do we integrate data from the community in the most seamless way possible?
What are the pros and cons r telehealth and related alternatives versus in-office visits?
How can we most effectively apply artificial intelligence to patient care?
How can we improve communication tools and information systems to support care anywhere?
How can we best integrate digital technologies to provide broad-based access?
How does VA Video Care work in select populations - older Veterans, rural Veterans, and women Veterans?
How can technology be used to care for the most vulnerable Veterans (older, rural, homeless, etc.)?
Do Veterans access or want to access health care resources through telehealth or technology-accessible programs?
How do we incorporate patient preferences in our questions about access to care?
Does increased access equal improvement, as demonstrated through better access, care quality, care coordination, patient satisfaction/preferences and manageable costs?
How do we most efficiently and appropriately match a patient with different access options given their clinical needs and preferences?
When Veterans have different options, what influences choosing VA?
What keeps Veterans engaged in care?
Why will some Veterans choose to 'never' come to the VA? What can VA do to bring down those barriers?
What do Veterans know about accessing/availability of healthcare?
How do we help Veterans understand their options for care, identify best option for them individually, then know how to access that care?
How can we determine and develop the right and appropriate metrics?
How can we determine the value and impact of our current access related measures?
How do we develop access metrics and criteria that reflect Veteran & Clinical needs?
How do we most efficiently and appropriately match a patient with different access options given their clinical needs and preferences?
How to measure perceived access to care?
Are the access metrics that we are using appropriate, and how do they need to be tailored for various conditions and needs (e.g., procedural versus medical)? Also, are they realistic compared to expectations in the community/private sector?
Who are we losing (i.e., who is not getting access), where are we losing them, and why?
What is the state and spread of provider availability and productivity?
How has full practice authority for APRNs affected access?
How can VA leverage tools & systems to increase clinician productivity as a means of improving access?
Can VA create systems to deliver the right care at the right time at a patient level?
How do we determine what the VA should build (provide) versus buy (community care)?
How can we improve staff/provider hiring and retention at the VA?
What novel care delivery methods improve access while maintaining quality?

Can activities such as peer-support, health coaching, whole health, and other activities supplement traditional care?

Appendix D. Final Access-Related Research Questions Ranked by Delphi Participants Through Rounds 2 and 3

Unique Research Questions (n=83)	Delphi panel Round 2 (n=18)	Delphi panel Round 3 (n=10)
How do we incorporate patient preferences in our questions about access to care?	✓	✓
How are the various dimensions (the 5 A's: affordability, availability, accessibility, accommodations, and acceptability) of access related to outcomes and value for Veterans?	✓	✓
How can we ensure equitable access to services for Veterans who are underrepresented or experience disparities in the VA?	✓	✓
Are there specific groups of enrolled Veterans who encounter barriers to care (e.g., language, literacy, transportation, lack of telehealth capacity, discrimination, harassment at facilities) and could targeted interventions enable these groups to overcome these barriers?	✓	✓
How do we determine what the VA builds (provides) or buys (community care)? What are ways to develop and enhance effective VA/community collaborative care models?	✓	✓
What are the unintended consequences of using telehealth to address access to care?	✓	✓
Does increased access equal improvement, as demonstrated through better access, care quality, care coordination, patient satisfaction/preferences and manageable costs?	✓	✓
How can non-face-to-face modalities be used to improve access?	✓	✓
What access barriers are interfering the most with Veterans getting the care they need?	✓	✓
How should "access" be defined and measured in the VA and in the community?	✓	✓
How can we design disease-specific and patient-risk-specific access measures to better represent the heterogeneity of access needs, rather than blanket designations?	✓	X
What are the pros versus cons of expanding services to access points in community partnership facilities (e.g., Walmart clinics, VERSUSO telehealth sites,	✓	X

libraries)? Is there an explicit need? If not, is there political or public relations value in creating them? Is there or will there be impact on enrollment or service usage?		
What are the best ways/metrics to measure Veteran access to care (e.g., survey items, admin data, important covariates)?	✓	X
How do we provide access to appropriate community services that are best delivered by the community and retain services best delivered by VA?	✓	X
How do we most efficiently and appropriately match a patient with different access options given their clinical needs and preferences?	✓	X
How can continuity of care best be maintained or improved when initiatives are introduced to improve access? For which VA patient groups is continuity of care most important?	✓	X
What other metrics or outcomes does access to care impact (e.g., trust, satisfaction, economic)?	✓	X
How can VA leverage tools and systems to increase clinician productivity as a means of improving access?	✓	X
What are the best overall metrics to determine "good access"?	X	X
What are the most important variables that should be used to measure access?	X	X
Does telehealth/virtual care exacerbate disparities?	X	X
What solutions for increasing access need to vary by subpopulation of Veterans (e.g., racial/ethnic minorities, LGBTQ, women, those living on tribal lands, etc.)?	X	X
What do vulnerable Veterans need to help them with access that is effective?	X	X
How will virtual care play a role in improving access in primary care in the future?	X	X
When can telehealth be used as a substitute for in-person care?	X	X

When does purchasing care in the community improve access to timely, high-quality care for Veterans?	X	X
What psychometrically validated instruments and methodologies exist (or can be developed) to measure Veterans' perceived access to care?	X	X
How can we improve actual and perceived access to care for patients who are struggling to manage chronic conditions?	X	X
How do we increase access to specialty care services without widening access disparities between urban/suburban and rural Veterans?	X	X
How do we improve access to evidence-based mental health care? How do we improve access to high quality guideline concordant mental health care provided in primary care? What novel care delivery methods improve access while maintaining quality?	X	X
How can we expand access to medical and social services through telemedicine and other technologies while ensuring that Veterans receive care that is at the same or higher quality than they would in person?	X	X
How do we optimize patient flows between primary and specialty care to manage overall access and quality of care?	X	X
How can we best support smooth transitions across community and VA care providers?	X	X
What novel care delivery methods improve access while maintaining quality?	X	X
What are the best approaches for understanding and managing Veterans' choices re: receiving care at VA versus. in the community?	X	X
What is the impact of the CHOICE and MISSION Acts in improving access and/or quality of care for Veterans?	X	X
How does VA Video Care work in select populations - older Veterans, rural Veterans, and women Veterans? What is the quality of care provided for these groups?	X	X

How can we improve access to gender-sensitive, integrated health care for women Veterans in the VA--and work with community partners to advance health care for women Veterans?	X	X
How has full practice authority for APRNs affected access?	X	X
What are the needs of our female Veteran population and is VHA appropriately preparing to provide care to that population group?	X	X
Are there specific groups of enrolled Veterans who have difficulty adhering to VA treatment recommendations and could improve access to care coordination services enable better adherence?	X	X
How do we best utilize technology to improve access to care (e.g., televideo, wearables)?	X	X
What are ways to decrease no-shows, make enrollment easier, and make access more efficient and easier for Veterans?	X	X
How do we best use administrative data to measure the access experience of patients receiving care through non-traditional channels, including virtual care, telephone care, secure messaging, and e-consults?	X	X
How can we leverage technology tools to improve access to timely care, especially mental health?	X	X
How can we address the acute shortage of mental health providers and care nationally?	X	X
How do we integrate data from the community in the most seamless way possible?	X	X
What are the pros and cons re: telehealth and related alternatives versus in-office visits?	X	X
How can we understand the tradeoffs and determine which services to support and under what conditions between VA and community care (including commercial retailers)?	X	X
How does virtual care impact access (i.e., how much does it augment versus. add?)	X	X
What does the intersection of virtual care and transportation look like? That is, given that we can	X	X

use virtual care for so much, does that obviate the necessity to expand rural transportation networks that are necessary to ensure access to face to face care? Or at least mitigate the effect of the current transportation deficit?		
What are Veteran perceptions about what is close/timely enough regarding care within VA and also in community care.	X	X
How can we decrease use of low-value services to improve access to high-value care?	X	X
How can we coordinate care and improve communication between VA and non-VA providers?	X	X
How can we ensure Veterans who are not currently accessing VA services due to distance from a VA facility, homelessness, or other factors are able to obtain quality care?	X	X
What are the needs of our aging Veteran population and is VHA appropriately preparing to provide care to that population group?	X	X
What are the best methods that could be routinely applied to VA data to measure continuity of care to ensure that continuity doesn't suffer when access is improved?	X	X
What are effective models to coordinate care received in VA and non-VA settings?	X	X
What do Veterans know about accessing/availability of healthcare?	X	X
How can we best make use of telemedicine to substitute for, rather than complement, in-person services?	X	X
How do we best match access interventions with Veteran needs, barriers, and resources?	X	X
How can we improve staff/provider hiring and retention at the VA?	X	X
How does closure/consolidation of Critical Access Hospitals affect Veteran Care?	X	X
Are there specific groups of eligible Veterans who are less likely to access VA services and could	X	X

targeted outreach to these groups ensure that they understand their options for accessing VA services?		
How can we improve access by leveraging social systems including family, friends, and community?	X	X
How to improve access to VA care for Veterans with visual impairment and for Veterans with visual dysfunction related to traumatic brain injury?	X	X
How can we optimize Veteran decision-making about use of VA and non-VA care? What are effective models to coordinate care received in VA and non-VA settings?	X	X
How does VA plan on mitigating the expected loss of roughly 60% of physicians and roughly 50% of APRNs as Primary Care providers over the next ten years?	X	X
What is the impact on access of provider burnout and/or interrupted continuity?	X	X
What is the correlation between resource utilization and outcomes in VA and non-VA settings?	X	X
Is there overdemand for certain clinical services that adversely affects access for those truly in need and, if so, how is overdemand defined?	X	X
How will Artificial Intelligence play a role in improving access in primary care in the future?	X	X
What are some of the most effective ways to reduce barriers to help seeking and retention for Veterans with health-related needs?	X	X
What processes and interventions can be reproduced and used in different contexts to understand and reduce barriers to access?	X	X
In what ways can VA support, and potentially engage in treatment, Veterans who do not or have not used VA recently?	X	X
What are factors that influence the metrics around drive times and wait times?	X	X
What is the state and spread of provider availability and productivity?	X	X

How will VA be able to hire qualified providers (physicians and APRNs) with current pay limitations?	X	X
Is VA a health care system or an insurance provider? And how does VA address the split between providing community care and care within VA itself?	X	X
What technology exists or should be created to aid in answering access-related questions and implementing the answers?	X	X
Do Veterans access (or want to access) health care resources through telehealth or technology-accessible programs?	X	X
When Medicare is expanded to everyone, what effect will that have on access and general system burden improvement?	X	X
How can we most effectively apply artificial intelligence to patient care?	X	X
